# Cold Atmospheric Plasma Promotes Killing of Staphylococcus aureus by Macrophages

**DOI:** 10.1128/mSphere.00217-21

**Published:** 2021-06-16

**Authors:** Constance Duchesne, Nadira Frescaline, Océane Blaise, Jean-Jacques Lataillade, Sébastien Banzet, Olivier Dussurget, Antoine Rousseau

**Affiliations:** a Institut de Recherche Biomédicale des Armées, INSERM UMRS-MD 1197, Centre de Transfusion Sanguine des Armées, Clamart, France; b Laboratoire de physique des plasmas, École Polytechnique, Sorbonne Université, CNRS, Palaiseau, France; c Institut Pasteur, Unité de Recherche Yersinia, Département de Microbiologie, Paris, France; d Université de Paris, Sorbonne Paris Cité, Paris, France; University of Rochester

**Keywords:** plasma jet, reactive oxygen species, reactive nitrogen species, staphylococci, phagosome maturation, LAMP-1, cathepsin D, *Staphylococcus aureus*, phagocyte

## Abstract

Macrophages are important immune cells that are involved in the elimination of microbial pathogens. Following host invasion, macrophages are recruited to the site of infection, where they launch antimicrobial defense mechanisms. Effective microbial clearance by macrophages depends on phagocytosis and phagolysosomal killing mediated by oxidative burst, acidification, and degradative enzymes. However, some pathogenic microorganisms, including some drug-resistant bacteria, have evolved sophisticated mechanisms to prevent phagocytosis or escape intracellular degradation. Cold atmospheric plasma (CAP) is an emerging technology with promising bactericidal effects. Here, we investigated the effect of CAP on Staphylococcus aureus phagocytosis by RAW 264.7 macrophage-like cells. We demonstrate that CAP treatment increases intracellular concentrations of reactive oxygen species (ROS) and nitric oxide and promotes the elimination of both antibiotic-sensitive and antibiotic-resistant S. aureus by RAW 264.7 cells. This effect was inhibited by antioxidants indicating that the bactericidal effect of CAP was mediated by oxidative killing of intracellular bacteria. Furthermore, we show that CAP promotes the association of S. aureus to lysosomal-associated membrane protein 1 (LAMP-1)-positive phagosomes, in which bacteria are exposed to low pH and cathepsin D hydrolase. Taken together, our results provide the first evidence that CAP activates defense mechanisms of macrophages, ultimately leading to bacterial elimination.

**IMPORTANCE** Staphylococcus aureus is the most frequent cause of skin and soft tissue infections. Treatment failures are increasingly common due to antibiotic resistance and the emergence of resistant strains. Macrophages participate in the first line of immune defense and are critical for coordinated defense against pathogenic bacteria. However, S. aureus has evolved sophisticated mechanisms to escape macrophage killing. In the quest to identify novel antimicrobial therapeutic approaches, we investigated the activity of cold atmospheric plasma (CAP) on macrophages infected with S. aureus. Here, we show that CAP treatment promotes macrophage ability to eliminate internalized bacteria. Importantly, CAP could trigger killing of both antibiotic-sensitive and antibiotic-resistant strains of S. aureus. While CAP did not affect the internalization capacity of macrophages, it increased oxidative-dependent bactericidal activity and promoted the formation of degradative phagosomes. Our study shows that CAP has beneficial effects on macrophage defense mechanisms and may potentially be useful in adjuvant antimicrobial therapies.

## INTRODUCTION

Macrophages are key components of the innate immune system. Along with neutrophils, they are in the first line of immune defense against infectious agents. In contrast to short-lived neutrophils, tissue macrophages are long-lived migratory cells that perform an array of immune surveillance functions and are equipped to recognize, phagocytose, and destroy microbial pathogens by oxidative and nonoxidative mechanisms (1–3). Pathogen recognition by phagocytic receptors triggers signaling cascades, leading to microbial uptake and formation of the early phagosome. Newly formed phagosomes are not initially microbicidal; a succession of fusion events between an intermediary phagosome and lysosomes results in the formation of mature phagolysosomes. During maturation, the phagosome gains microbicidal activity with progressive acidification and acquisition and activation of multiple antimicrobial components such as lactoferrin, NADPH oxidase, lysozyme, lipases, and proteases (including cathepsins) (4). This dynamic process is characterized by the appearance and disappearance of membrane proteins through successive fusion and fission between phagosomes and early endosomes, late endosomes, and, finally, lysosomes. New phagosomes rapidly acquire early endosome proteins such as early endocytic antigen 1 (EEA1) and the small GTPase Rab5. Further phagosome maturation leads to progressive loss of Rab5, recruitment of Rab7 mediating fusion with late endosomes, and acquisition of lysosomal-associated membrane proteins (LAMPs) (5).

Despite the potency of macrophage antimicrobial arsenal, some pathogenic bacteria have developed strategies to preclude pathogen degradation (6). For instance, some intracellular pathogens prevent phagosome-lysosome fusion, while others modify the intraphagosomal environment or escape into the cytosol (7). Increasing evidence suggests that Staphylococcus aureus, an important human pathogen that causes a wide range of clinical infections (8), has evolved mechanisms to evade intracellular defense mechanisms that may lead to replication within macrophages (9). Infected macrophages may form reservoirs that lead to bacterial persistence and cause recurrence of infection (10). Furthermore, S. aureus is notorious for its ability to become resistant to antibiotics, which poses a great challenge for infection control by limiting treatment options (11, 12).

Cold atmospheric plasma (CAP) refers to partially ionized gases that are globally neutral, since the density of positively charged species is equivalent to that of the negatively charged species. CAP is produced by electrical discharge in a neutral gas (see Fig. S1 in the supplemental material). Due to their high reactivity and temperature close to room temperature, cold plasmas are appropriate for biological applications (13). Cold plasmas are known to produce thermal radiation, reactive radicals, ions and electrons, visible light, UV radiation, and electromagnetic fields. Their interactions with ambient air lead to the production of reactive species that can induce multiple biological effects. Chemical species produced at significant concentrations include reactive oxygen and nitrogen species (RONS) such as ozone, superoxide anion, and hydrogen peroxide, and nitrogen oxides (NOx), including nitrite, nitrate and nitric oxide (Fig. S1). Previous studies showed that low CAP energy deposition has stimulating effects on the proliferation and migration of cells, whereas high-energy deposition induces lethal effects that are studied for cancer treatment (14–16). We recently reported that treatment with CAP promotes cutaneous tissue repair (14). In addition to its prohealing properties, CAP has previously been shown to have antimicrobial properties (17–23). The mode of action of CAP is not completely understood; however, it is reasonable to speculate that RONS, which are generated by CAP, may have an important role in enhancing host immune response, leading to reduced survival of pathogenic bacteria. Thus, we hypothesized that CAP could stimulate elimination of bacteria by macrophages. In this study, we demonstrate that CAP treatment is associated with enhanced killing of both methicillin-sensitive S. aureus (MSSA) and methicillin-resistant S. aureus (MRSA) strains following phagocytosis by macrophages. This effect is mediated by oxidative mechanisms, since antioxidants inhibit CAP-dependent bactericidal activity of macrophages. Furthermore, CAP treatment promotes phagosome maturation into a degradative vesicle. Our study uncovers the ability of CAP to enhance phagosomal defense mechanisms in infected macrophages.

10.1128/mSphere.00217-21.1 FIG S1 Generation and biological effects of cold atmospheric plasma (CAP). CAP is partially ionized gas that is produced by providing energy (high voltage) to a neutral gas via electrodes. Insulators are placed between electrodes and air to keep a low current and prevent the creation of an electric arc. Helium can be used as a carrier gas to propagate the discharge on several centimeters. CAP is composed of physical components and reactive oxygen species (ROS) and reactive nitrogen species (RNS) in liquid and gas phases, such as ozone, nitrite, nitrate, nitric oxide, and hydrogen peroxide. O°, oxygen radical; NO, nitric oxide; OH°, hydroxyl radical; NO_2_^−^, nitrite; NO_3_^−^, nitrate; O_3_, ozone; H_2_O_2_, hydrogen peroxide; H_2_O, water; O2°^−^, superoxide anion. Download FIG S1, EPS file, 1.4 MB. Copyright © 2021 Duchesne et al. 2021 Duchesne et al. https://creativecommons.org/licenses/by/4.0/ This content is distributed under the terms of the Creative Commons Attribution 4.0 International license.

## RESULTS

### CAP treatment is associated with increased intracellular levels of reactive oxygen species and NO in macrophages.

**FIG 1:**
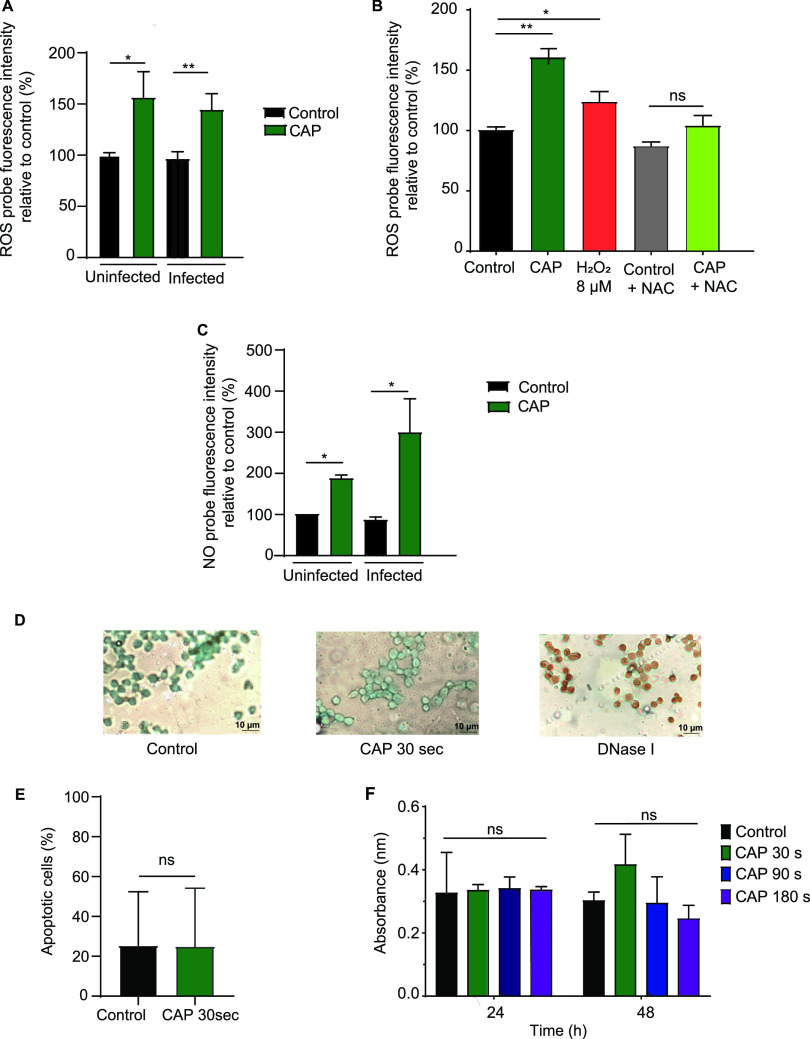
Cold atmospheric plasma (CAP) treatment increases the levels of reactive oxygen species (ROS) and nitric oxide (NO) in macrophages without altering their viability. (A to C) RAW 264.7 macrophages were left uninfected or were infected with Staphylococcus aureus Xen36 for 1 h, and nonengulfed bacteria were killed using gentamicin for a 30-min period before treatment with CAP. (A) RAW 264.7 macrophages were incubated with 20 μM fluorogenic 2,7-dichlorodihydrofluorescein diacetate (DCFH-DA) to quantify intracellular ROS. Relative fluorescence of cells untreated (control) or treated with CAP for 30 s was measured 45 min after treatment. Data are mean ± standard error of the mean (SEM) (*, *P* < 0.05; **, *P* < 0.01, ns, nonsignificant; one-way analysis of variance [ANOVA] followed by Dunn’s posttest; *n* = 9). (B) RAW 264.7 macrophages were incubated with 20 μM DCFH-DA for 45 min to quantify intracellular ROS. Relative fluorescence of uninfected cells treated with 8 μM H_2_O_2_, or with CAP for 30 s, with or without 1 mM *N*-acetyl cysteine (NAC), was measured using a fluorescence microplate reader. Data are mean ± SEM (*, *P* < 0.05; **, *P* < 0.01, ns, nonsignificant; one-way ANOVA followed by Dunn’s posttest; *n* = 3). (C) RAW 264.7 macrophages were incubated with 5 mM fluorescent 4-amino-5-methylamino-2′,7′-diacetate fluorescein (DAF-FM DA) for 1 h to quantify the level of intracellular NO. Relative fluorescence of cells untreated (control) and cells treated with CAP for 30 s was measured by flow cytometry. Data are mean ± SEM (*, *P* < 0.05, ns, nonsignificant; one-way ANOVA followed by Dunn’s posttest; *n* = 9). (D) Representative images of terminal deoxynucleotidyltransferase-mediated dUTP-biotin nick end labeling (TUNEL) staining of RAW 264.7 cells left untreated (control), 24 h after CAP treatment for 30 s or after treatment with 1 μg/μL DNase I, used as a positive control. Brown nuclear staining with 3,3′-diaminobenzidine (DAB) indicates DNA damage. DNA was counterstained with methyl green. (E) Quantitative detection of apoptosis by TUNEL assay at 24 h posttreatment. Data are mean ± SEM (Student’s *t* test; *n* = 3; ns, nonsignificant). (F) Analysis of cellular viability using the WST-1 assay on RAW 264.7 cells left untreated (control), or at 24 and 48 h after CAP treatment for 30, 90, or 180 s. Data are mean ± SEM (Mann-Whitney U test; *n* = 6; ns, nonsignificant).

To investigate if CAP could modulate macrophage defenses, we measured the intracellular RONS levels upon CAP treatment of RAW 264.7 cells infected or not with S. aureus. We first determined the levels of intracellular reactive oxygen species (ROS) by measuring 2,7-dichlorodihydrofluorescein diacetate (DCFH-DA) fluorescence in macrophages following CAP treatment. CAP induced a significant increase in intracellular ROS levels in both infected and noninfected macrophages compared to those in untreated macrophages (*P* < 0.05 and *P* < 0.01, respectively; one-way analysis of variance [ANOVA] followed by Dunn’s posttest; *n* = 9) (Fig. 1A). As expected, ROS levels did not increase when cells were incubated with antioxidant *N*-acetyl cysteine (NAC) prior to CAP treatment (one-way ANOVA followed by Dunn’s posttest; *n* = 3) (Fig. 1B). Higher levels of intracellular ROS were also detected upon 8 μM H_2_O_2_ treatment compared to those in untreated macrophages, which corresponds to the concentration of H_2_O_2_ produced by the CAP treatment under our experimental conditions (*P* < 0.05; one-way ANOVA followed by Dunn’s posttest; *n* = 3) (Fig. 1B). Interestingly, ROS levels were lower than those upon CAP treatment, unless a much higher concentration of H_2_O_2_ was used (Fig. S2A). We next determined the levels of nitric oxide (NO) by measuring 4-amino-5-methylamino-2′,7′-diacetate fluorescein (DAF-FM DA) fluorescence in macrophages following CAP treatment. The levels of intracellular NO were significantly higher in macrophages treated by CAP than those in untreated macrophages, irrespective of S. aureus infection (*P* < 0.05; one-way ANOVA followed by Dunn’s posttest; *n* = 9) (Fig. 1C). As expected, NO levels were also increased in macrophages treated with the NO donor *S*-nitroso-*N*-acetyl-dl-penicillamine (SNAP) (see Fig. S2B in the supplemental material). To determine if CAP treatment was toxic to cells, we measured the apoptosis and the viability of RAW 264.7 macrophages. We first evaluated apoptosis levels of macrophages by terminal deoxynucleotidyltransferase-mediated dUTP-biotin nick end labeling (TUNEL) assay upon CAP treatment. Untreated macrophages and CAP treated-macrophages showed similarly low levels of apoptosis by TUNEL staining (Student’s *t* test; *n* = 3) (Fig. 1D and E). In contrast, DNase I treatment, as expected, led to massive DNA damage (Fig. 1D). We next determined cellular viability using the water-soluble tetrazolium salt 1 (WST-1) assay at 24 and 48 h after CAP treatment. Untreated macrophages and macrophages treated with CAP for 30 s had similar viabilities at both time points (Mann-Whitney U test; *n* = 6) (Fig. 1F). Increasing the treatment duration to 90 or 180 s did not lead to any significant increase in cell mortality. Together, our results indicate that macrophage intracellular ROS and NO levels are increased upon CAP treatment without any significant cellular toxicity.

10.1128/mSphere.00217-21.2 FIG S2 Effect of reactive oxygen and nitrogen species (RONS) on the levels of ROS in macrophages. (A) RAW 264.7 cells were treated with 100 μM H_2_O_2_ as an ROS positive control. Values are means of fluorescence intensity relative to the control ± standard error of the mean (SEM) (**, *P* < 0.01; Mann-Whitney U test; *n* = 3). (B) RAW 264.7 cells were treated with 45 μM *S*-nitroso-*N*-acetyl-dl-penicillamine (SNAP) as an RNS positive control. Values are means of fluorescence intensity relative to the control ± SEM (**, *P* < 0.01; Mann-Whitney U test; *n* = 3). Download FIG S2, EPS file, 1.5 MB. Copyright © 2021 Duchesne et al. 2021 Duchesne et al. https://creativecommons.org/licenses/by/4.0/ This content is distributed under the terms of the Creative Commons Attribution 4.0 International license.

### CAP treatment increases S. aureus killing by macrophages in an ROS-dependent manner.

**FIG 2:**
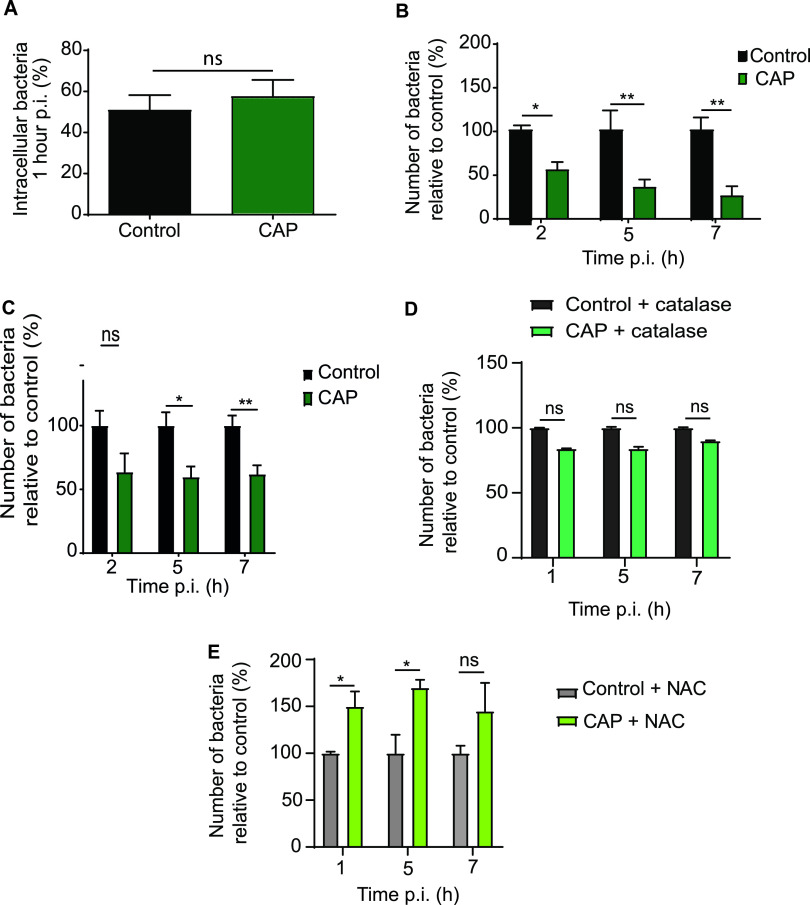
CAP treatment promotes elimination of S. aureus in macrophages. RAW 264.7 macrophages were treated with CAP or left untreated (control) in 24-well plates at a voltage of 32 kV, a gap of 5 mm, and a helium mass flow of 500 sccm. After incubation overnight, cells were infected with S. aureus Xen36 or USA300 strains. (A) Relative number of S. aureus Xen36 contained in macrophages 1 h postinfection, corresponding to the ratio of the number of internalized bacteria to the total number of bacteria used to infect cells. (B) Number of S. aureus Xen36 bacteria contained in macrophages at 2, 5, and 7 h postinfection (h p.i.) after CAP treatment relative to the number of bacteria contained in untreated macrophages (*, *P* < 0.05; **, *P* < 0.01; ****; *P* < 0.0001, CAP versus control; 2-way ANOVA followed by Tukey’s posttest). Three independent experiments were performed with 4 to 6 replicates per conditions. (C) Number of S. aureus USA300 bacteria contained in macrophages at 2, 5, and 7 h postinfection after CAP treatment relative to the number of bacteria contained in untreated macrophages (*, *P* < 0.05; **, *P* < 0.01, CAP versus control; 2-way ANOVA followed by Tukey’s posttest; *n* = 3). (D) Number of S. aureus Xen36 bacteria contained in macrophages at 1, 5, and 7 h postinfection after CAP treatment relative to the number of bacteria contained in untreated macrophages in the presence of 0.1 mg/ml catalase. (E) Number of S. aureus Xen36 bacteria contained in macrophages at 1, 5, and 7 h postinfection after CAP treatment relative to the number of bacteria contained in untreated macrophages in the presence of 1 mM NAC (*, *P* < 0.05, CAP plus NAC versus NAC only; 2-way ANOVA followed by Tukey’s posttest; *n* = 5).

Having demonstrated that CAP affects macrophage defenses, we next investigated if CAP could increase the bactericidal activity of macrophages. A gentamicin protection assay was used to evaluate the impact of CAP treatment on phagocytosis and intracellular bacterial survival. Phagocytosis of S. aureus by RAW 264.7 macrophages was very efficient at 37°C (Fig. 2A), while it was expectedly blocked at 4°C (see Fig. S3 in the supplemental material), irrespective of treatment. The percentage of internalized bacteria was similar in CAP-treated and untreated macrophages at 1 h postinfection (Fig. 2A). Thus, CAP treatment does not significantly influence the capacity of macrophages to internalize S. aureus. However, the number of viable intracellular bacteria was significantly lower in CAP-treated macrophages than those in untreated macrophages at 2, 5, and 7 h postinfection (h p.i.) (*P* < 0.05 at 2 h p.i. and *P* < 0.01 at 5 and 7 h p.i.; 2-way ANOVA followed by Tukey’s posttest; *n* = 4) (Fig. 2B; see also Fig. S4 in the supplemental material). We next wondered if CAP could also enhance killing by macrophages of an antibiotic-resistant strain of S. aureus, the MRSA strain USA300. The percentage of intracellular bacteria was lower in macrophages treated by CAP than that in untreated macrophages (*P* < 0.05 at 5 h p.i. and *P* < 0.01 at 7 h p.i.; 2-way ANOVA followed by a Tukey’s posttest; *n* = 3) (Fig. 2C). Thus, CAP treatment promotes killing of both MSSA and MRSA strains by macrophages. These effects were not due to enhanced gentamicin permeability following CAP treatment because the intracellular concentration of gentamicin was similar in macrophages treated with CAP and in untreated cells (see Fig. S5 in the supplemental material). Additionally, intracellular gentamicin concentration stayed under its MIC for S. aureus Xen36 (i.e., 8 μg/ml), irrespective of treatment, even when the protection assay was performed with 50 μg/ml of gentamicin (Fig. S5). Furthermore, CAP treatment did not increase cell death or proliferation, as the number of macrophages did not differ between conditions (Fig. 1D to F). Along these lines, levels of macrophage cell surface and intracellular markers, including those of growth factors and cytokines, remained unchanged in response to CAP treatment (see Fig. S6A and S6B in the supplemental material). Since we observed a link between CAP treatment and ROS production by macrophages, we next investigated the role of oxidative defenses in CAP-dependent killing of S. aureus. Bacterial survival was quantified in macrophages treated or not with CAP and in the presence of catalase and NAC. Catalase inhibited the effect of CAP on bacterial killing, and the combination of CAP and NAC even increased the number of bacteria (*P* < 0.05; 2-way ANOVA followed by Tukey’s posttest; *n* = 5) (Fig. 2D and E). It is of note that direct CAP treatment of S. aureus can also induce the death of bacteria in an ROS-dependent manner (*P* < 0.01; one-way ANOVA followed by Dunn’s posttest; *n* = 6 to 9) (see Fig. S7A and B in the supplemental material). Together, our results show that CAP increases the bactericidal activity of macrophages in an ROS-dependent manner, without affecting their capacity to internalize bacteria.

10.1128/mSphere.00217-21.3 FIG S3 Effect of CAP treatment on S. aureus phagocytosis by macrophages. RAW 264.7 cells were infected with S. aureus Xen36 and incubated for 1 h at 37°C or 4°C. The number of internalized bacteria was determined after cell lysis by plating serial dilutions on agar (**, *P* ≤ 0.01; 2-way analysis of variance [ANOVA] followed by Tukey’s posttest; *n* = 3). Download FIG S3, EPS file, 1.7 MB. Copyright © 2021 Duchesne et al. 2021 Duchesne et al. https://creativecommons.org/licenses/by/4.0/ This content is distributed under the terms of the Creative Commons Attribution 4.0 International license.

10.1128/mSphere.00217-21.4 FIG S4 CAP treatment promotes S. aureus killing in macrophages. CFU of S. aureus Xen36 contained in macrophages at 2, 5, and 7 h postinfection, untreated (control) or after CAP treatment (*, *P* < 0.05; **, *P* < 0.01; ****, *P* < 0.0001, CAP versus control; 2-way ANOVA followed by Tukey’s posttest, *n* = 4). Download FIG S4, EPS file, 1.2 MB. Copyright © 2021 Duchesne et al. 2021 Duchesne et al. https://creativecommons.org/licenses/by/4.0/ This content is distributed under the terms of the Creative Commons Attribution 4.0 International license.

10.1128/mSphere.00217-21.5 FIG S5 Effect of CAP treatment on gentamicin internalization by macrophages. RAW 264.7 cells were treated with CAP or left untreated (control) and incubated for 24 h after treatment in fluorescent gentamicin-containing medium. The quantity of internalized gentamicin was determined by enzyme-limited immunosorbent assay (ELISA) after 0, 3, and 6 h of incubation (2-way ANOVA followed by Tukey’s posttest; *n* = 3; ns, nonsignificant). Download FIG S5, EPS file, 1.3 MB. Copyright © 2021 Duchesne et al. 2021 Duchesne et al. https://creativecommons.org/licenses/by/4.0/ This content is distributed under the terms of the Creative Commons Attribution 4.0 International license.

10.1128/mSphere.00217-21.6 FIG S6 Effect of CAP treatment on levels of macrophage cell surface markers, growth factors, and cytokines. RAW 264.7 cells were treated with CAP or left untreated (control). After 24 h of incubation at 37°C, half of the wells were infected with S. aureus Xen36 at a multiplicity of infection (MOI) of 10. Protein expression was quantified using flow cytometry. (A) Levels of cell surface markers without infection and at 1 and 6 h postinfection (p.i.) (Kruskall-Wallis test; ns, nonsignificant; *n* = 6). (B) Levels of cytokines and growth factor, without infection and at 1 and 6 h postinfection (Kruskall-Wallis test; ns, nonsignificant; *n* = 9). Download FIG S6, EPS file, 1.6 MB. Copyright © 2021 Duchesne et al. 2021 Duchesne et al. https://creativecommons.org/licenses/by/4.0/ This content is distributed under the terms of the Creative Commons Attribution 4.0 International license.

10.1128/mSphere.00217-21.7 FIG S7 CAP treatment triggers S. aureus killing in an ROS-dependent manner. S. aureus Xen36 was grown in brain heart infusion (BHI), and 1 × 10^6^ bacteria/ml were treated with CAP at a voltage of 32 kV, a gap of 5 mm, and helium mass flow of 500 standard cm^3^/min (sccm). (A) A 2-ml bacterial suspension was treated for 1, 2 or 3 min. (B) A 2-ml bacterial suspension was treated with CAP for 1 min, with or without 1 mM *N*-acetyl cysteine (NAC) or 0.1 mg/ml catalase (CAT). CFU were determined by plating serial dilutions on BHI agar. Data are mean ± SEM (*, *P* < 0.05; **P < 0.01 versus control; one-way ANOVA followed by the Dunn’s posttest; *n* = 6 to 9). Download FIG S7, EPS file, 2 MB. Copyright © 2021 Duchesne et al. 2021 Duchesne et al. https://creativecommons.org/licenses/by/4.0/ This content is distributed under the terms of the Creative Commons Attribution 4.0 International license.

### CAP treatment is associated with increased numbers of acidic, LAMP-1-, and cathepsin D-positive vesicles in macrophages infected with S. aureus.

**FIG 3:**
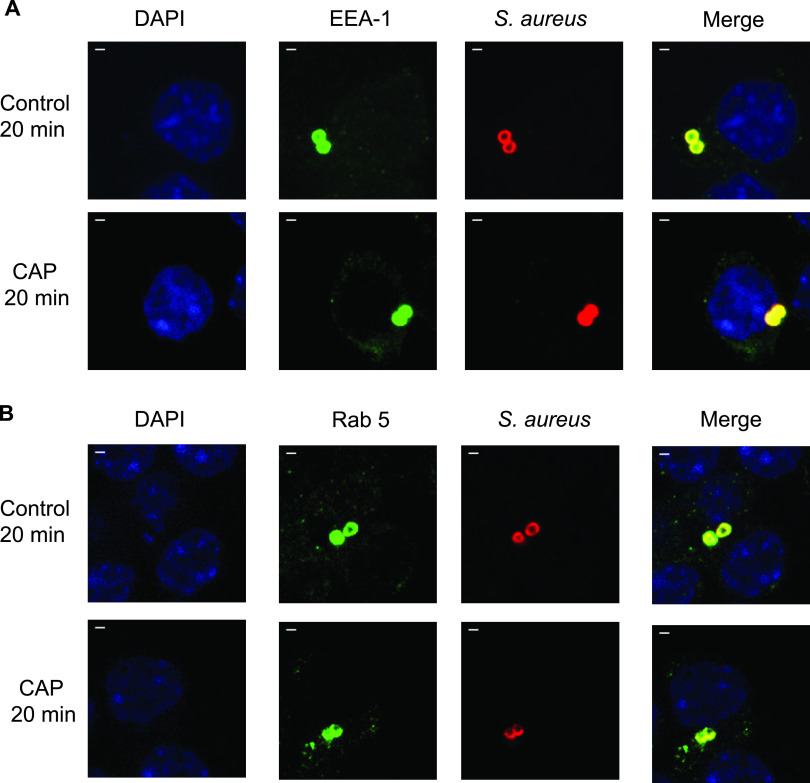
CAP treatment does not influence the acquisition of early phagosomal markers in macrophages infected with S. aureus. CAP-treated or untreated RAW 264.7 macrophages were infected with S. aureus Xen36 (multiplicity of infection [MOI] = 10), and markers of early phagosomes were analyzed by immunofluorescence at 20 min postinfection. (A) Representative confocal images of EEA-1 staining. EEA-1 (green), bacteria (red), nucleus (blue), EEA-1/bacteria colocalization (yellow). DAPI, 4′,6-diamidino-2-phenylindole. (B) Representative confocal images of Rab5 staining. Rab5 (green), bacteria (red), nucleus (blue), Rab5/bacteria colocalization (yellow). Bar, 5 μm.

**FIG 4:**
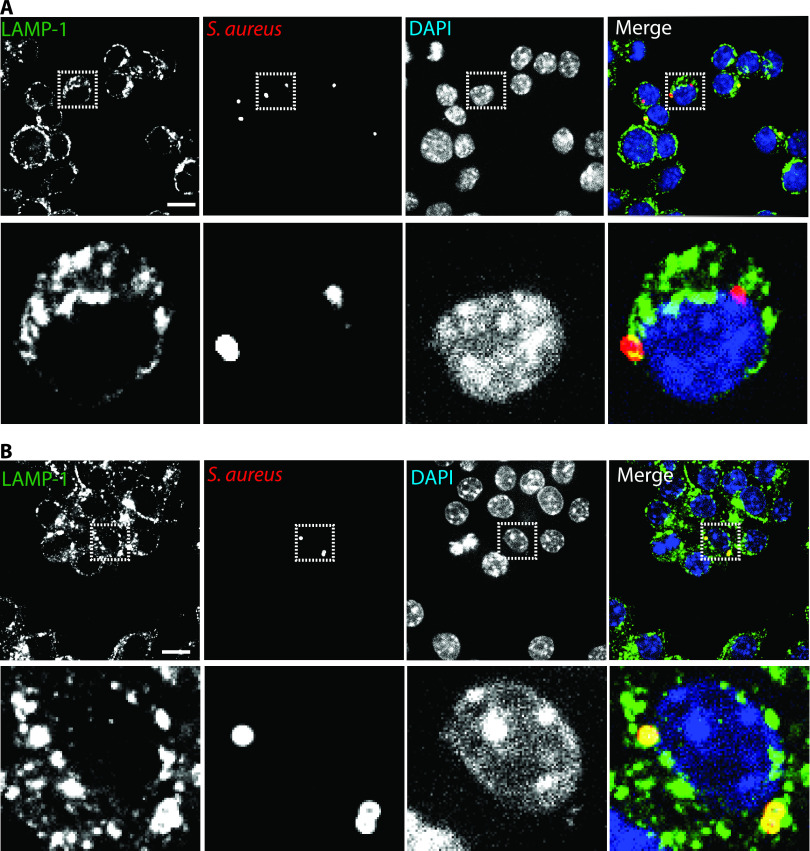
CAP treatment promotes the recruitment of LAMP-1, a late phagosomal marker, in macrophages infected with S. aureus. CAP-treated and untreated RAW 264.7 cells were infected with S. aureus Xen36 (MOI = 10), and LAMP-1 recruitment was analyzed by immunofluorescence at 6 h postinfection. Representative confocal images of LAMP-1 staining in untreated RAW 264.7 cells (A) and in CAP-treated RAW 264.7 cells (B). LAMP-1 (green), bacteria (red), nucleus (blue), LAMP-1/bacteria colocalization (yellow). Upper panels are ×63 magnification; bar, 20 μm. Lower panels represent enlarged views of the boxed regions.

**FIG 5:**
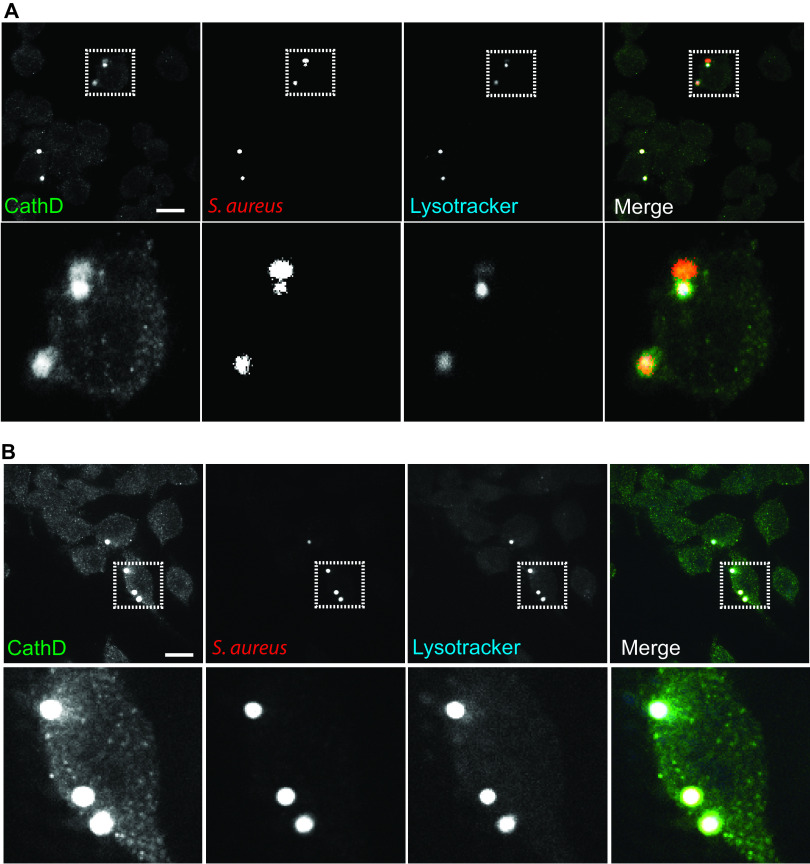
CAP treatment is associated with recruitment of cathepsin D and with phagosome acidification in macrophages infected with S. aureus. CAP-treated and untreated RAW 264.7 cells were infected with S. aureus Xen36 (MOI = 10). Cathepsin D recruitment and phagosomal acidification were analyzed by immunofluorescence at 6 h postinfection. Representative images of cathepsin D staining and LysoTracker signal in untreated RAW 264.7 cells (A) and in CAP-treated RAW 264.7 cells (B). Cathepsin D (green), LysoTracker (blue), bacteria (red), cathepsin D/bacteria colocalization (yellow), cathepsin D/LysoTracker/bacteria colocalization (white). Upper panels are ×63 magnification; bar, 20 μm. Lower panels represent enlarged views of the boxed regions.

**FIG 6:**
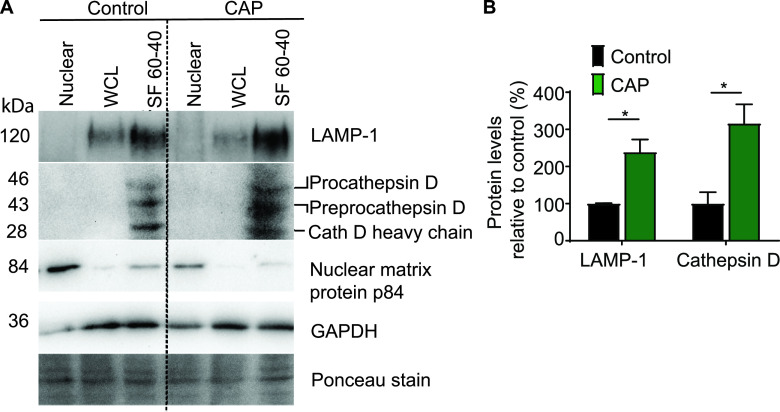
CAP treatment increases the levels of phagosomal LAMP-1 and cathepsin D in macrophages infected with S. aureus. Untreated (control) and CAP-treated RAW 264.7 cells were infected with S. aureus Xen36 (MOI = 10) and submitted to subcellular fractionation at 6 h postinfection. (A) Western blot analysis of nuclear fraction, whole-cell lysate (WCL), and subcellular fraction 60 to 40% (SF 60-40) of control (left) and CAP-treated (right) cells upon sucrose gradient ultracentrifugation. The same amount of total protein (40 μg) was analyzed for each fraction. Samples were immunoblotted with anti-LAMP-1, anti-cathepsin D, anti-nuclear matrix protein p84, and anti-GAPDH antibodies. Ponceau staining of polyvinylidene difluoride (PVDF) blotting membrane was used as a loading control. LAMP-1, lysosomal-associated membrane protein 1; GADPH, glyceraldehyde 3-phosphate dehydrogenase. (B) Relative levels of LAMP-1 and cathepsin D in SF 60-40 of S. aureus-infected RAW 264.7 cells. Values are means ± SEM of three replicates (*, *P* < 0.05, Student’s *t* test; *n* = 3).

Having established that CAP stimulates bactericidal activity of macrophages by oxidative mechanisms, we next wondered whether CAP had additional effects on macrophage defense mechanisms. We therefore analyzed the phagosomal maturation upon CAP treatment of macrophages infected with S. aureus. Bacteria and markers of early (EEA1 and Rab5) and late (LAMP-1 and cathepsin D) phagosomes were detected by immunofluorescence at 20 min, 1 h, and 6 h postinfection. After 20 min of infection, S. aureus was detected in EEA1-positive phagosomes (Fig. 3A) and in Rab5-positive phagosomes (Fig. 3B) of both CAP-treated and untreated macrophages. At that time point, more than 70% of S. aureus-containing phagosomes were positive for EEA1 and Rab5, while only ∼10% were positive for LAMP-1 (Table 1). As expected, the percentage of bacteria associated with early phagosomal markers decreased with time (Table 1). CAP treatment did not lead to any significant change in the number of early phagosomes associated with S. aureus (Mann-Whitney U test) (Table 1). The number of bacteria associated with late phagosomal markers progressively increased over time (Table 1). By 1 h postinfection, the number of LAMP-1-positive phagosome containing bacteria increased to 25%. At 6 h postinfection, the percentage of LAMP-1-positive S. aureus-containing phagosomes reached 36% in untreated macrophages (Table 1). Strikingly, the number of LAMP-1-positive phagosomes containing bacteria was higher in macrophages treated with CAP than that in untreated macrophages (Fig. 4). The percentage of phagosomes showing an association between S. aureus and LAMP-1 increased to 46% upon CAP treatment (*P* < 0.05, Mann-Whitney U test) (Table 1). To confirm that CAP modulated late stages of phagosome maturation, we next imaged and quantified the association of the lysosomal enzyme cathepsin D with phagosomes containing S. aureus. At 1 h after infection, a third of the phagosomes were positive for cathepsin D, irrespective of CAP treatment (Table 1). After 6 h of infection, S. aureus was also detected in cathepsin D-positive phagosomes of both CAP-treated and untreated macrophages (Fig. 5). However, the percentage of S. aureus-containing phagosomes that were positive for cathepsin D was higher in CAP-treated macrophages than that in untreated macrophages, i.e., 53 and 43%, respectively (*P* < 0.01, Mann-Whitney U test) (Table 1). Interestingly, S. aureus localized to cathepsin D-positive acidic phagosomes (Fig. 5), and phagosomal acidification was promoted by CAP treatment (*P* < 0.001, Mann-Whitney U test) (Table 1). To confirm our quantification based on microscopic observations, we determined the levels of markers on phagosomes isolated from infected macrophages upon CAP treatment. To verify the purity of isolated phagosomes, known cellular organelle markers were probed. Nuclear matrix protein p84 was mostly detected in the nuclear fraction, and the ubiquitous GAPDH was detected in all fractions, while LAMP-1 and cathepsin D were clearly only present in the phagosomal fraction (Fig. 6A). Western blot analysis demonstrated that CAP treatment significantly increased the levels of both LAMP-1 and cathepsin D in the 60 to 40% fractions of S. aureus-infected macrophages by 6 h postinfection (*P* < 0.05, Student’s *t* test; *n* = 3) (Fig. 6B). Together, these results suggest that CAP treatment promotes the formation of degradative organelles upon phagocytosis of S. aureus.

**TABLE 1:** Percentage of colocalization of S. aureus Xen36 bacteria with phagosome markers in RAW 264.7 cells untreated (control) and treated with CAP

Phagosome marker	Colocalization % at time p.i.^*a*^:					
	20 min		1 h		6 h	
	Control	CAP	Control	CAP	Control	CAP
EEA-1	79.8 ± 1.8	77.5 ± 1.3	48.3 ± 3.8	45.9 ± 3.4	26.2 ± 2.9	30.7 ± 2.6
Rab5	72.0 ± 1.5	70.4 ± 1.1	51.4 ± 2.1	50.4 ± 2.2	16.0 ± 0.6	16.1 ± 0.8
LAMP-1	12.1 ± 1.4	10.6 ± 0.7	25.1 ± 1.7	29.5 ± 3.4	36.0 ± 2.8	46.5 ± 2.5^*b*^
Cathepsin D	ND	ND	36.6 ± 5.7	31.6 ± 3.4	43.8 ± 1.5	53.6 ± 1.4^*c*^
Lysotracker	ND	ND	37.5 ± 5.2	28.7 ± 4.3	44.3 ± 2.7	61.1 ± 2.2^*c*^

a Mean value ± standard error of the mean (SEM). ND, not determined; p.i., postinfection.

b *P* < 0.05; Mann-Whitney U test.

c *P* < 0.001; Mann-Whitney U test.

## DISCUSSION

The ability of some pathogenic bacteria to escape immune defense mechanisms and the emergence of multidrug resistance pose a significant threat to human health (24). Unprecedented global spread of antibiotic-resistant bacteria, coupled with a low number of preclinical and clinical antibacterial drugs in the pipeline, highlights the urgent need for the development of pathogen-specific and adjunctive antimicrobial approaches (25). S. aureus has been listed by the World Health Organization in the ESKAPE (Enterococcus faecium, Staphylococcus aureus, Klebsiella pneumoniae, Acinetobacter baumannii, Pseudomonas aeruginosa, and Enterobacter species) group of bacteria against which new therapeutics are urgently required (26). S. aureus displays an arsenal of virulence factors that allows it to persist intracellularly within macrophages by impairing host cell defenses such as phagosomal acidification, phagosome maturation, and cathepsin D activation (27). In this study, we investigated the effect of CAP on the phagocytic activity of RAW 264.7 macrophages infected with S. aureus. While CAP treatment did not influence the ability of macrophages to engulf bacteria, it significantly increased the bactericidal activity of infected macrophages. Interestingly, killing of both MSSA and MRSA strains was increased by CAP. This finding is significant because the clinical MRSA strain USA300 that was used in this study is highly virulent and has been shown to survive in macrophages by perturbing phagolysosome formation (28). Given that CAP has previously been shown to induce changes in cellular membrane permeability (29, 30), we wondered if the increase in bacterial killing could be due to elevated intracellular gentamicin concentration induced by CAP. This hypothesis was refuted by our data, which clearly demonstrated that CAP did not change the intracellular concentration of gentamicin, which remained at subinhibitory levels. Our results suggest that the increased bacterial killing associated with CAP was not due to changes in cellular viability either.

CAP is a source of a wide variety of potentially bactericidal ROS, such as hydrogen peroxide, superoxide, hydroxyl radicals, and ozone, and of reactive nitrogen species (RNS), including nitrite, nitrate and NO (31). Likewise, phagocyte activation results in the production of intracellular ROS derived from NADPH oxidase (NOX2) and of RNS, which are generated as a result of enzymatic activity of inducible nitric oxide synthase (iNOS/NOS2) (32). Robust production of RONS is critical for the elimination of microbial pathogens (33–39). Thus, we hypothesized that oxidative mechanisms could contribute to bacterial killing by macrophages treated with CAP. We demonstrated that the action of CAP was ROS dependent because dismutation of hydrogen peroxide by catalase and ROS scavenging by NAC inhibited CAP-dependent bacterial killing by macrophages. NAC even promoted S. aureus survival after CAP treatment, possibly through redox control of cellular defense components and/or bacterial factors. Moreover, CAP treatment rapidly increased intracellular ROS concentration in macrophages. Upon CAP treatment, bacterial killing by macrophages could rely on both endogenous and exogenous ROS (40). However, in our experimental design, macrophages were treated 24 h before the infection. This design does not support a direct bactericidal role for CAP-generated exogenous ROS, and it can reasonably be hypothesized that macrophage endogenous ROS were increased upon CAP treatment, leading to increased bactericidal effects. We also demonstrated that the intracellular concentration of NO in macrophages increases immediately after CAP treatment. NO possesses important immunologic functions (41–43) and reacts with superoxide anion (O_2_°^−^) to produce peroxynitrite, a powerful oxidant capable of inactivating intramacrophagic microorganisms (44). Thus, the capacity of CAP to rapidly deliver and promote formation of both ROS and RNS in macrophages possibly contributes to overcoming bacterial immune escape strategies and to controlling infection. The composition of CAP is complex, and its physical components (e.g., UV and electromagnetic fields) may have a biological activity independently of and/or in synergy with its chemical components. When exposed to CAP, bacteria can, for instance, be altered by UV radiation and electromagnetic fields (45–47). In macrophages, NADPH oxidase generates O_2_°^−^ through electron transfer from cytosolic NADPH to oxygen, producing electron current (48). Therefore, it is reasonable to hypothesize that electric fields generated by CAP contribute to the modulation and activation of the phagocyte NADPH oxidase complex.

Oxidative killing in macrophages occurs in a dynamic process of maturation of the phagosome. During phagosomal maturation, macrophages also eliminate phagocytosed microbes by nonoxidative mechanisms, such as acidification and proteolytic degradation along phagosome maturation pathways (5). We hypothesized that CAP could modulate the phagosome maturation of infected macrophages. CAP treatment did not affect the early stages of phagosome maturation. Indeed, EEA-1- and Rab5-positive compartments progressed to LAMP-1-positive compartments with rapid loss of both EEA1 and Rab5 in late phagosomes by 6 h postinfection, independently of CAP treatment. In contrast, CAP promoted association of LAMP-1 and cathepsin D with phagosomes containing S. aureus. These results suggest that CAP treatment favors the maturation of phagosomes into phagolysosomes. The colocalization of S. aureus and cathepsin D at 6 h postinfection occurred in acidic phagosomes, which promote the activity of hydrolases. Furthermore, the localization of S. aureus within acidic phagosomes was significantly increased upon CAP treatment. The molecular mechanisms underlying the effect of CAP on phagosome maturation remain to be determined. CAP could possibly promote phagosomal maturation through upregulation of intracellular ROS production, as has recently been shown for bioactive lipid lysophosphatidylcholine (49). In conclusion, we have shown here that CAP enhances macrophage defense mechanisms upon S. aureus infection. As the capacity of S. aureus to survive intracellularly contributes to its pathogenicity, CAP is an attractive adjuvant therapeutic candidate that could be used in the health care setting to control staphylococcal infections.

## MATERIALS AND METHODS

### Cold atmospheric plasma.

CAP was generated using a device that we previously described (15). Briefly, CAP was generated in a dielectric polylactic acid (PLA) capillary flown with helium and using a high-voltage electrode surrounding the capillary. CAP was propagated from the source to the target in the form of a single-channel plasma jet. RONS are formed at the output of the capillary when plasma interacts with ambient air. High voltage was set to 32 kV, and helium mass flow was set to 500 sccm, leading to a power of 80 ± 10 W. Production of long-lived species in liquid exposed to CAP increased proportionally with treatment time. The concentrations of H_2_O_2_, NO_2_^−^, and NO_3_^−^ that corresponded to a 30-s CAP treatment were 8, 1.2, and 0.5 μM, respectively.

### Bacteria and growth conditions.

S. aureus Xen36, an MSSA strain derived from a clinical isolate of a bacteremic patient, was obtained from PerkinElmer (Villebon-sur-Yvette, France). S. aureus USA300, a highly virulent MRSA strain involved in multiple outbreaks in healthy individuals, was obtained from ATCC (reference BAA-1717). Bacteria were grown aerobically at 37°C in brain heart infusion (BHI) broth (BD Biosciences, San Jose, CA) with shaking at 200 rpm or on BHI agar plates.

### Cell line and culture conditions.

The murine macrophage-like cell line RAW 264.7 (ATCC, Molsheim, France) was grown in RPMI medium (Gibco, Paisley, UK) supplemented with 4 mM GlutaMax and 1 mM sodium pyruvate (Thermo Fisher Scientific, Waltham, MA) and 10% fetal bovine serum (BioWest, Nuaillé, France) at 37°C in 10% CO_2_ atmosphere.

### Quantification of ROS levels in macrophages.

RAW 264.7 cells were seeded onto 24-well plates at a density of 10^5^ cells per well. RAW 264.7 cells were left uninfected or were infected with S. aureus Xen36 at a multiplicity of infection (MOI) of 10:1 for 1 h. Cells were incubated in 20 μg/ml gentamicin-containing medium for 30 min before treatment with CAP to kill extracellular bacteria. They were then incubated with the cell-permeant fluorogenic ROS probe DCFH-DA (Sigma-Aldrich Ltd., Dublin, Ireland) at a final concentration of 20 μM for 5 min at 37°C. Cells were treated with CAP or H_2_O_2_ (Sigma-Aldrich, St. Louis, MO) at a concentration corresponding to the one produced by our CAP treatment conditions (8 μM). CAP treatment was performed in culture medium or in culture medium supplemented with 1 mM NAC (Sigma-Aldrich, St. Louis, MO). After 45 min of incubation, cells were resuspended in phosphate-buffered saline (PBS). The suspension was transferred into a 96-well microplate, and fluorescence was measured using a Synergy HT multimode microplate reader (BioTek Instruments, Winooski, VT) at excitation and emission wavelengths of 485 nm and 525 nm, respectively.

### Quantification of NO levels in macrophages.

RAW 264.7 cells were seeded onto 24-well plates at a density of 10^5^ cells per well. RAW 264.7 cells were left uninfected or were infected with S. aureus Xen36 (MOI = 10) for 1 h. Cells were incubated in 20 μg/ml gentamicin-containing medium for 30 min before treatment with CAP to kill extracellular bacteria. They were then incubated with the cell-permeant fluorescent NO probe DAF-FM DA (Sigma, Saint-Quentin-Fallavier, France) at a final concentration of 5 mM for 5 min at 37°C before treatment. A solution of the NO donor SNAP (Sigma-Aldrich, St. Louis, MO) at 45 μM was used as a positive control. After 1 h of incubation at 37°C, cells were resuspended in PBS, and fluorescence was measured by flow cytometry at 500 nm (CytoFlex S; Beckmann-Coulter, Villepinte, France).

### Quantification of bacteria survival in macrophages.

RAW 264.7 cells were seeded onto 24-well plates at a density of 10^5^ cells per well. Cells were treated with CAP for 30 s in fresh culture medium or were left untreated, then incubated at 37°C overnight. For inhibition assays, 1 mM NAC (Sigma-Aldrich, St. Louis, MO) or 0.1 mg/ml catalase (Sigma-Aldrich, St. Louis, MO) were added to the culture medium before CAP treatment. Cells were infected with S. aureus at an MOI of 10:1 and incubated for 1 h at 37°C to allow phagocytosis or at 4°C to block phagocytosis. To eliminate extracellular bacteria, cells were incubated in 20 μg/ml gentamicin-containing medium. Infected cells were lysed at 1, 2, 5, and 7 h postinfection with 0.2% Triton X-100 for 5 min, and dilution series were plated onto BHI agar for enumeration of intracellular bacteria and quantification of bacterial CFU.

### Quantification of intracellular gentamicin.

RAW 264.7 cells were seeded onto 24-well plates at a density of 10^5^ cells per well. Cells were then treated with CAP for 30 s in fresh culture medium or left untreated (control), then incubated at 37°C overnight. Cells were infected with S. aureus at an MOI of 10:1 and incubated at 37°C for 1 h to allow phagocytosis. Cells were incubated in 0, 5, 20, or 50 μg/ml gentamicin-containing culture medium. After 0, 3, and 6 h of incubation, cells were lysed and gentamicin concentration determined using a gentamicin enzyme-limited immunosorbent assay (ELISA) kit (Creative Diagnostics, Shirley, NY) according to the instructions of the suppliers.

### Quantification of cellular viability and proliferation.

Cellular viability was determined using the stable tetrazolium salt WST-1 reagent (Sigma-Aldrich, St. Louis, MO), which is cleaved to soluble formazan by cellular mitochondrial dehydrogenases. RAW 264.7 cells were treated with CAP for 30 s, and the WST-1 assay was conducted according to the manufacturer’s instructions at 24 h and 48 h posttreatment. Absorbance was measured using a microplate reader (Labsystems, Finland) at 450 nm.

### TUNEL assay.

A TUNEL assay (Sigma-Aldrich, St. Louis, MO) was conducted to detect apoptotic cells undergoing extensive DNA degradation. RAW 264.7 cells were treated with CAP for 30 s and incubated for 24 h before TUNEL staining according to the manufacturer’s instructions. DNase I (Sigma-Aldrich, Saint-Louis, MO) at 1 μg/μl in 1× Tris-buffered saline (TBS)/1 mM MgSO_4_ was used as a positive control. At the end of the assay, coverslips were mounted on microscope slides and analyzed using an inverted microscope (Eclipse TE2000-U; Nikon, Tokyo, Japan) equipped with a high-resolution digital camera (ORCA-ER; Hamamatsu Photonics, Tokyo, Japan). Images were analyzed by manual counting of labeled cells. For each point, at least 100 S. aureus-containing vacuoles were counted and scored for the presence or absence of markers. Ten fields of microscopic view per well were captured and averaged. Three independent experiments were performed with 3 replicates per experiment.

### Antibodies.

Goat anti-EEA1 (catalog no. ab206860), rat monoclonal anti-LAMP-1 (catalog no. ab25245), mouse monoclonal anti-nuclear matrix protein p84 (catalog no. ab487), and rabbit anti-Staphylococcus aureus (catalog no. ab20920) antibodies were obtained from Abcam (Cambridge, UK). Goat anti-cathepsin D (catalog no. STJ140018) and goat anti-Rab5b (catalog no. STJ140061) antibodies were purchased from St John’s Laboratory (London, UK). Donkey anti-goat IgG (H+L) cross-adsorbed Alexa 488 (catalog no. A-11055), goat anti-rat IgG (H+L) cross-adsorbed Alexa 488 (catalog no. A110006), and goat anti-rabbit IgG (H+L) highly cross-adsorbed Alexa 488 (catalog no. A-11034) secondary antibodies were purchased from Invitrogen (Thermo Fisher Scientific). Goat anti-rabbit IgG (H+L) Cy3 and goat anti-rabbit IgG (H+L) C5 antibodies were purchased from Jackson ImmunoResearch Europe Ltd. (Cambridge, UK). For Western blotting, mouse monoclonal anti-LAMP-1 (catalog no. 611043) were purchased from BD sciences (San Jose, CA), mouse monoclonal anti-GAPDH, (catalog no. 60004-1) was purchased from Proteintech (Manchester, UK), nuclear matrix protein p84 (catalog no. ab487) was purchased from Abcam (Cambridge, UK), mouse anti-rabbit horseradish peroxidase (HRP; catalog no. SC-2357), goat anti-mouse IgG HRP (catalog no. SC-2005), and mouse anti-goat IgG HRP (catalog no. SC-2354) were purchased from Santa Cruz Biotechnology (Santa Cruz, CA). For flow cytometry, rat monoclonal anti-mouse CD80 fluorescein isothiocyanate (FITC; catalog no. 13365564), anti-mouse CD86 phycoerythrin (PE)-cyanine 7 (catalog no. 15598436), anti-mouse CD206 PE (catalog no. 16360604), anti-mouse CD163 allophycocyanin (APC; catalog no. 16380684), anti-mouse transforming growth factor beta (TGF-β; catalog no. 15558676), anti-mouse interleukin 10 (IL-10) FITC (catalog no. 15298349), anti-mouse tumor necrosis factor alpha (TNF-α) PE (catalog no. 15259319), and anti-mouse IL-1β APC (catalog no. 15350830) and the corresponding IgG isotype controls were purchased from Thermo Fisher Scientific.

### Flow cytometry.

RAW 264.7 cells were seeded onto 24-well culture plates at 5 × 10^5^ cells per well. After 24 h, cells were treated with brefeldin A (5 μg/ml) to increase intracellular staining. Cells were harvested at 1 h, 6 h, and 24 h after treatment and washed twice with cold phosphate-buffered saline (PBS). For surface staining, cells were incubated with 20 μl of FITC-conjugated anti-CD80, PE-conjugated anti-CD206, APC-conjugated anti-CD86, PE-Cy7-conjugated anti-CD167, or isotype-matched controls for 20 min on ice. For cytokine staining, cells were incubated with 20 μl of FITC-conjugated anti-IL-10, PE-conjugated anti-TNF-α, APC-conjugated anti-IL-1-β PE-Cy7-conjugated anti-TGF-β, or isotype-matched controls for 20 min on ice. Then, stained cells were suspended in cold buffer and analyzed by flow cytometry (CytoFlex; Beckman Coulter, Villepinte, France).

### Fluorescence microscopy.

RAW 264.7 cells were seeded onto glass coverslips at a density of 10^5^ cell/cm^2^. Cells were either treated with CAP for 30 s or left untreated, then incubated at 37°C overnight. Cells were then infected with S. aureus Xen36 at an MOI of 10:1. Three different time points were studied. One group of cells were fixed for 15 min with 4% paraformaldehyde (PFA; Electron Microscopy Sciences, Hatfield, PA) at 20 min postinfection. The second group of cells were incubated in culture medium containing 20 μg/ml of gentamicin at 30 min postinfection and fixed with 4% PFA at 1 or 6 h postinfection. Fixed cells were permeabilized with 0.1% Triton TX-100 (Sigma-Aldrich, Saint-Louis, MO) and blocked with 5% goat serum (Sigma-Aldrich, Saint-Louis, MO). Cells were then incubated with primary antibodies for 1 h, and then with fluorophore-tagged secondary antibodies for 1 h. Finally, 1 μM 4′,6-diamidino-2-phenylindole (DAPI) was added to cells for 15 min. Samples were mounted on glass coverslips and observed with a Zeiss Axio Observer Z1 confocal microscope (Zeiss, Oberkochen, Germany). Analyses were performed with CellProfiler software (50). For each point, at least 100 S. aureus*-*containing phagosomes were counted and scored for the presence or absence of markers. Results were expressed as the mean percentage of marker colocalization on S. aureus-containing phagosomes plus or minus the standard error of the mean (SEM). To study acidic compartments, LysoTracker Red DND-99 (Fisher Scientific, Illkirch, France) was added along with bacteria. The LysoTracker Red DND-99 was used concomitantly with a cathepsin D marker to quantify phagosomal acidification. The anti-S. aureus antibody enables the detection of both dead and living bacteria.

### Cell fractionation.

RAW 264.7 cells were fractionated according to a previously described protocol (51). Briefly, cells were washed and resuspended in PBS-bovine serum albumin (BSA) 0.5%, and then centrifuged at 300 × *g* for 5 min. PBS-BSA 0.5% was exchanged for homogenization buffer (8% sucrose in imidazole 3 mM, MgCl_2_ 1 mM supplemented with EGTA 0.5 mM, gelatin 0.5%, and Complete protease inhibitors [Roche, Mannheim, Germany]) and cells were centrifuged at 300 × *g* for 10 min. Cells were then resuspended and mechanically disrupted in homogenization buffer using a 25-gauge 5/8 needle. After centrifugation at 2,000 × *g* for 15 min, the postnuclear fraction was collected, brought to 40%, sucrose and loaded on top of a 60% sucrose cushion. A discontinuous 60/40%, 40/30%, 30/20%, and 20/8% sucrose gradient was prepared and ultracentrifuged at 100,000 × *g* for 1 h. The recovered fractions were adjusted to a final concentration of 10% sucrose.

### Immunoblotting.

Cellular and subcellular extracts from RAW 264.7 cells were lysed in ice-cold radioimmunoprecipitation assay (RIPA) buffer (Cell Signaling Technology, Beverly, MA) supplemented with Complete protease inhibitors (Roche). The lysate was centrifuged at 15,000 × *g* for 10 min at 4°C, and the supernatant was assayed for protein content. Protein content was quantified using a Pierce BCA bicinchoninic acid (BCA) quantification kit (Thermo Fisher Scientific, Rockford, IL) according to the manufacturer's instructions. Total protein was prepared in Laemmli buffer (Bio-Rad Laboratories, Hercules, CA), and 40 μg of protein was subjected to SDS-PAGE separation and immunoblotting. Following protein electrotransfer, a polyvinylidene difluoride (PVDF) membrane (Immun-Blot; Bio-Rad Laboratories) was incubated in Ponceau S staining solution for 5 to 10 min at room temperature. An image of the Ponceau-stained membrane was captured. Membranes were incubated with primary antibodies directed against LAMP-1 (1:100 dilution), cathepsin D antibody (1:200 dilution), GAPDH (1:2,000 dilution), and nuclear matrix protein p84 (1:500 dilution), and revealed with secondary antibodies (1:2,000 dilution). Chemiluminescent detection was performed using the Clarity Western ECL substrate (Bio-Rad Laboratories). Chemiluminescence signals were acquired using the ChemiDoc X-ray spectrometry plus (XRS+) system and Image Lab software (Bio-Rad Laboratories). Signal intensity was determined by densitometry to calculate relative levels of protein.

### Statistical analysis.

Statistical analysis was performed using Prism 6.04 software (GraphPad Software, San Diego, CA). For a two-group comparison, a nonparametric Mann-Whitney U test was used. For a comparison of more than two groups, a nonparametric Kruskall-Wallis test (one-way ANOVA) was used. In this case, multiple comparisons were done with Dunn’s test. When two variables were studied, a two-way ANOVA was used with Tukey’s multiple-comparison posttest to compare replicate means. In this case, it was assumed that the distribution of the sample was normal. Data were expressed as mean values ± SEM, and *P* values lower than 0.05 (*), 0.01 (**), or 0.001 (***) were considered to be statistically significant.
